# Supplementary material for: Phytocannabinoids Reduce Inflammation of Primed Macrophages and Enteric Glial Cells: An In Vitro Study
Source: Int J Mol Sci. 2023 Sep 27;24(19):14628. doi: 10.3390/ijms241914628 (PMC10572654; doi:10.3390/ijms241914628)
Supplement: Supplementary file 1 [file ijms-24-14628-s001.zip › ijms-2575166-supplementary.pdf]

Supplemental Figures, Table and Sequencing information

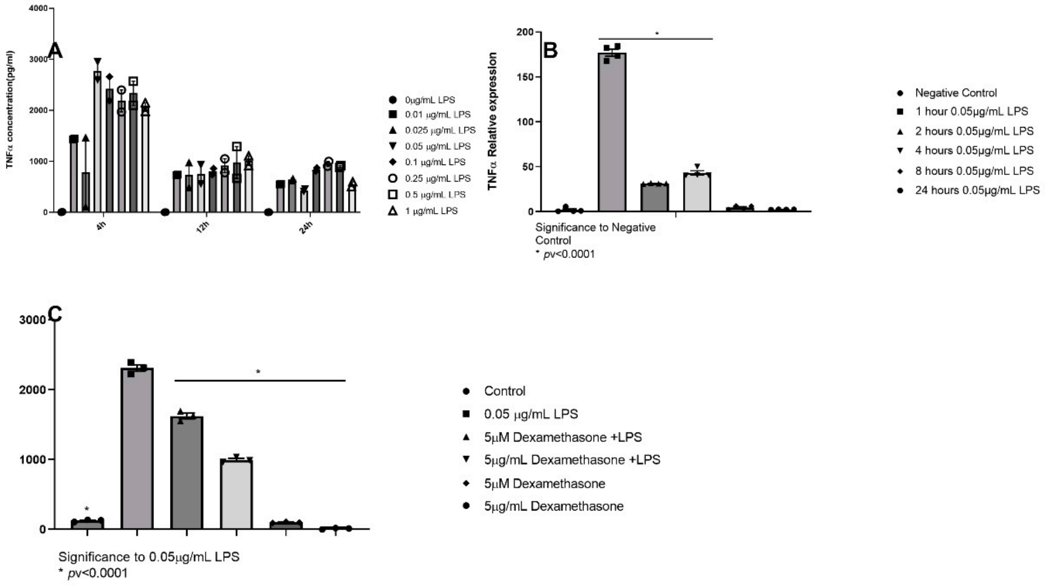

Figure S1: Calibration of maximal LPS induced TNFα secretion and expression in J77A1 macrophages and maximal reduction of TNFα by dexamethasone. Macrophages were incubated with different concentration of LPS and media was removed for analysis at different times. A. 0.01-1μg/mL LPS at 4,12 and 24h, B. TNFα relative expression with 0.05μg/mL at 1, 2, 4, 8, 24h, measured by RTqPCR. C. concentration of 5μM and 5μg/mL were added to cells with or w/o incubation of 4h with LPS. n=4, all samples were compared to positive control Dunnett's multiple comparison test.

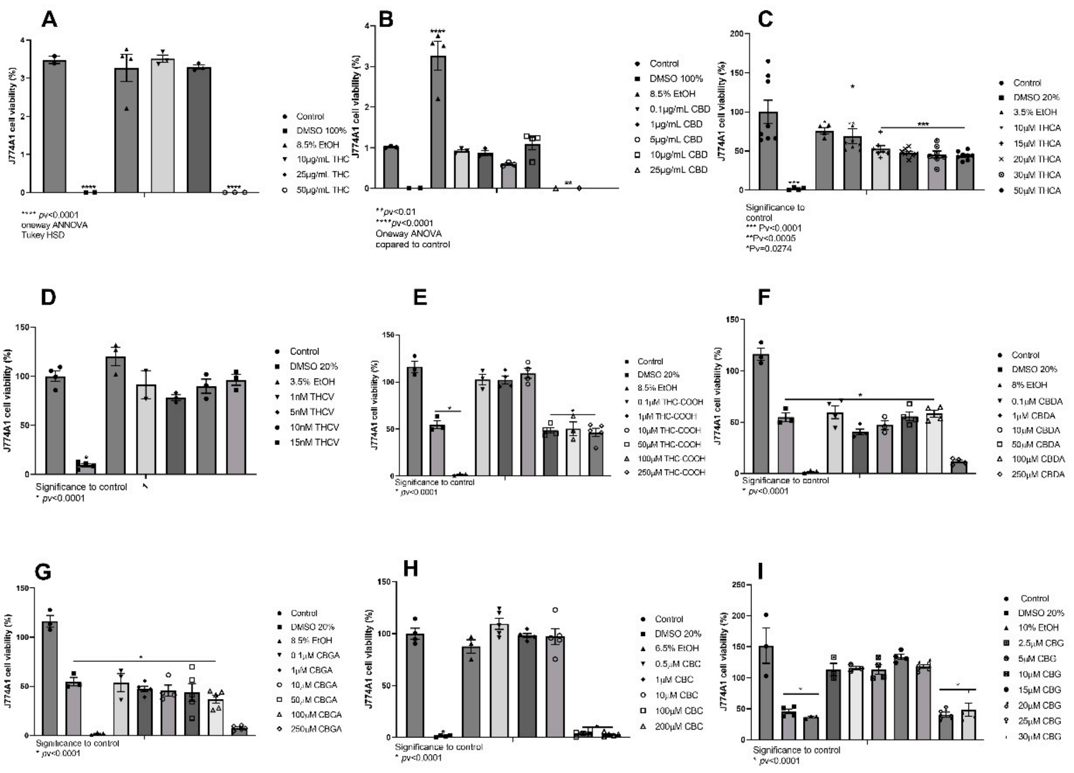

Figure S2: Cell viability on J774A1 cells were incubated with medium containing synthetic cannabinoids for 24h and then placed for 2h with 0.5mg/mL MTT 3-(4,5-dimethylthiazol-2-yl)-2,5-diphenyltetrazolium bromide. Non-treated cells served as positive control 100% DMSO-negative control, all cannabinoids were dissolved in EtOH, EtOH was added to volume equivalent to the highest cannabinoid. Absorbance was read at 550nm. A. 10-50µg/mL THC, B. 0.1-25µg/mL CBD, C. 10-50µM THCA, D. 1-15nM THCV, E. 0.1-250µM THC-COOH, F. 0.1-250µM CBDA, G. 0.1-250µM CBGA, H. 0.05-200µM CBC, I. 2.5-30µM CBG. n=4, all samples were compared to positive control Dunnett's multiple comparison test.

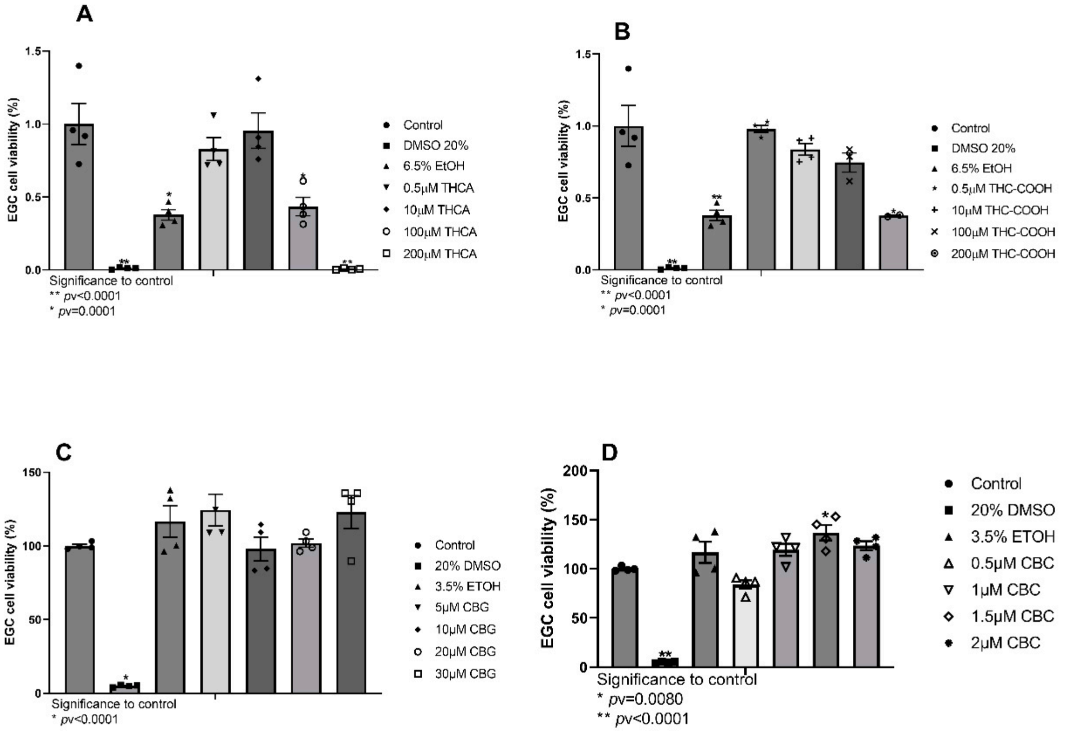

Figure S3: Cell viability on EGCs were incubated with medium containing synthetic cannabinoids for 24h and then placed for 2h with 0.5mg/mL MTT 3-(4,5-dimethylthiazol-2-yl)-2,5-diphenyltetrazolium bromide. Non-treated cells served as positive control 100% DMSO-negative control, all cannabinoids were dissolved in EtOH, EtOH was added to volume equivalent to the highest cannabinoid. A. 0.5-200μM THCA, B. 0.5-200μM THC-COOH, C. 5-30μM CBG, D. 0.5-2μM CBC. . n=4, all samples were compared to positive control Dunnett's multiple comparison test.

To summarize toxicity on both J774A1 murine macrophages and rat EGC by MTT assay [46] (Fig S2 and Fig S3). For J774A1 macrophages THC was not toxic in the range of 0.5-10μg/mL, CBD 10-50μM, THCA 1-20μM, THC-COOH 0.1-50μM, CBC 1-10μM, CBG 2.5-20μM, THCV 1-15nM, CBD 1-10μg/mL, CBDA, CBGA solution significantly decreased J774A1 cell viability more than 50% at all the concentrations that were tested (Fig S2),. Cell viability was not decreased in EGC at the following concentrations; THCA 0.5-10 μM, THC-COOH 0.5-100μM, CBG 5-30μM, CBC 0.5-2μM.

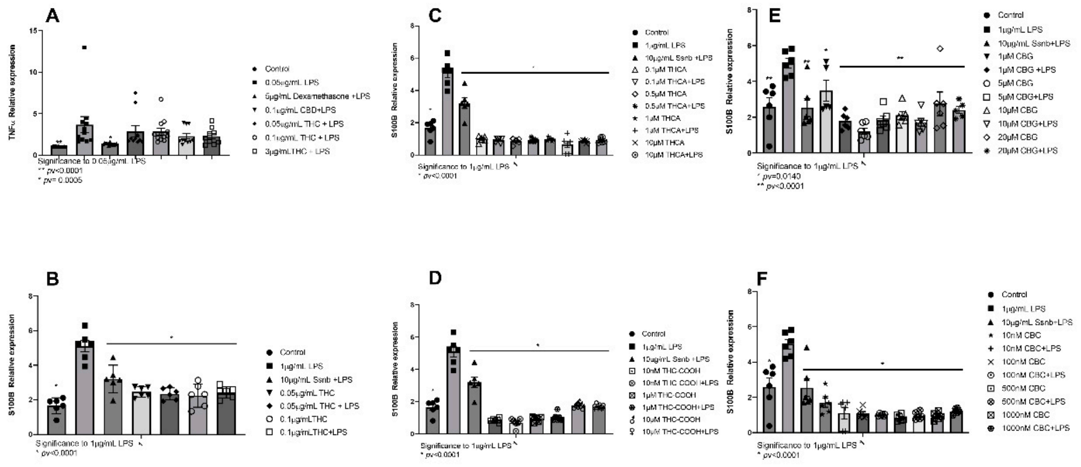

Figure S4: J774A1 and EGC were incubated for 1h with synthetic cannabinoids and then with 0.05μg/mL LPS for J774A1 and 0.1μg/mL for EGC Dexamethasone was used as positive control for J774A1 cells and Ssnb for EGC. Relative expression was measured using RTqPCR. A. Relative expression of Tnfα gene after incubation with 0.1-3μg/mL THC and 1μg/mL LPS, B. Relative expression of S100B gene after incubation with 0.05-0.1μg/mL THC with and w/o 1μg/mL LPS. C. Relative expression of S100B after incubation with 0.01-10μM THCA with and w/o 1μg/mL LPS. D. Relative expression of S100B after incubation with 10nM-10μM of THC-COOH with or w/o 1μg/mL LPS. E. Relative expression of S100B after incubation with 1-20μM of CBG with or w/o 1μg/mL LPS. F. Relative expression of S100B after incubation with 10-1000nM CBC with or w/o 1μg/mL LPS. A n=12, B-F n=6. All samples were compared to positive control Dunnett's multiple comparison test.

Table S1: Primers used for RTqPCR

Name	Forward	Reverse	Accession
m-PPIA	GGGTTCCTCCTTTCACAGAA	GATGCCAGGACCTGTATGCT	NM_008907
m-TNFα	GTCTGTGCCTCAGCCTCTTC	GCTTGGTGGTTTGCTACGAC	U68414.1
r-GAPDH	TGAGGTGACCGCATCTTCTTG	TGGTAACCAGGCGTCCGATA	AF106860.2
r- S100B	CAGGAGCCTCCGGATGT	TCCTGCTCTTTGATTTCCTCCA	NM_013191.2
r-GFAP	TCCTGGAACAGCAAAACAAG	CAGCCTCAGGTTGGTTTCAT	Z48978.1

All sequencing data (GEO Submission (GSE240225) [NCBI tracking system #24220033] is available at https://www.ncbi.nlm.nih.gov/geo/query/acc.cgi?acc=GSE240225
